# Valorization of Sugarcane Bagasse for Co-Production of Poly(3-hydroxybutyrate) and Bacteriocin Using *Bacillus cereus* Strain S356

**DOI:** 10.3390/polym16142015

**Published:** 2024-07-15

**Authors:** Sunisa Khamberk, Sutticha Na-Ranong Thammasittirong, Anon Thammasittirong

**Affiliations:** 1 Department of Science and Bioinnovation, Faculty of Liberal Arts and Science, Kasetsart University, Nakhon Pathom 73140, Thailandsutticha.n@ku.ac.th (S.N.-R.T.); 2 Microbial Biotechnology Unit, Faculty of Liberal Arts and Science, Kasetsart University, Nakhon Pathom 73140, Thailand

**Keywords:** *Bacillus*, bacteriocin, bioplastic, lignocellulose, polyhydroxyalkanoate, poly(3-hydroxybutyrate), sugarcane bagasse

## Abstract

Poly(3-hydroxybutyrate) (P(3HB)) is an attractive biodegradable plastic alternative to petroleum-based plastic. However, the cost of microbial-based bioplastic production mainly lies in the cultivation medium. In this study, we screened the isolates capable of synthesizing P(3HB) using sugarcane bagasse (SCB) waste as a carbon source from 79 *Bacillus* isolates that had previously shown P(3HB) production using a commercial medium. The results revealed that isolate S356, identified as *Bacillus cereus* using 16S rDNA and *gyrB* gene analysis, had the highest P(3HB) accumulation. The highest P(3HB) yield (5.16 g/L, 85.3% of dry cell weight) was achieved by cultivating *B. cereus* S356 in an optimal medium with 1.5% total reducing sugar with SCB hydrolysate as the carbon source and 0.25% yeast extract as the nitrogen source. Transmission electron microscopy analysis showed the accumulation of approximately 3–5 P(3HB) granules in each *B. cereus* S356 cell. Proton nuclear magnetic resonance spectroscopy and Fourier transform infrared spectroscopy analyses confirmed that the polymer extracted from *B. cereus* S356 was P(3HB). Notably, during cultivation for P(3HB) plastic production, *B. cereus* S356 also secreted bacteriocin, which had high antibacterial activity against the same species (*Bacillus cereus*). Overall, this work demonstrated the possibility of co-producing eco-friendly biodegradable plastic P(3HB) and bacteriocin from renewable resources using the potential of *B. cereus* S356.

## 1. Introduction

Petroleum-based plastics are promising materials for many applications due to their properties and low price. However, the non-biodegradable property of conventional plastics results in their accumulation in the environment and toxicity to ecosystems. In 2021, global production of plastics reached 390.7 million tons, with 90.2%, 8.3%, and 1.5% being produced from fossil-based, recycled, and bio-based polymers, respectively [1]. Biodegradable plastic is an effective option that has gained attention in the past two decades to reduce the utilization of petroleum-derived plastics. Several types of biodegradable plastics have been studied. Among them, there has been increasing attention given to poly(3-hydroxybutyrate) (P(3HB)), the most frequently occurring and studied member of the polyhydroxyalkanoate (PHA) bioplastic family due to its properties that resemble those of conventional petroleum-based plastics, such as polypropylene (PP) and polyethylene (PE). In addition, it is completely biodegradable and is seen as an ideal alternative to conventional plastics in several applications such as packaging, agriculture, pharmacology, and medical devices [2,3]. However, high cost is a major obstacle in the large-scale production of P(3HB) bioplastic, resulting in it commanding market prices that are six times higher than conventional petroleum-based plastics [4]. P(3HB) is synthesized and kept intracellularly as granules for energy storage and is found in various microorganisms during growth under stress conditions of nutrient imbalance [2,5]. In addition to energy storage, recent studies have reported the secondary function of P(3HB) in protecting cells from thermal and oxidative stresses (for a review see [6]). P(3HB) has been reported to be synthesized by various species of more than 55 genera of Gram-positive and Gram-negative bacteria such as *Bacillus* spp., *Brevibacterium casei*, *Burkholderia thailandensis*, *Cupriavidus necator*, *Pseudomonas* spp., and *Ralstonia eutropha*. [2,7,8]. However, P(3HB) produced from Gram-positive bacteria, which lack outer membrane lipopolysaccharide (LPS) that may co-purify with P(3HB), was reported to be more competitive for biomedical applications [9]. Currently, P(3HB) production on a commercial scale is based on high-cost feedstocks such as purified sugars. The high cost of the carbon source for bacterial fermentation is a major factor that accounts for approximately 50% of the entire production cost [10,11]. Several research studies have attempted to investigate the utilization of lower-cost carbon sources, especially wastes and byproducts from several industries [7,9].

Lignocellulosic biomass (LCB) from agricultural crops and agroindustry wastes is an attractive material for use as feedstock in P(3HB) production [7,9]. Sugarcane is one of the most important foods and industrial crops cultivated globally, providing approximately 85% of the world’s sugar. According to a report by the Food and Agricultural Organization of the United Nations, global sugarcane cultivation surpassed 1.9 billion tons in 2022, with the top five sugarcane-producing countries being Brazil, India, China, Thailand, and Pakistan [12]. Sugarcane bagasse (SCB) is a lignocellulosic waste from the sugar industry, with approximately 0.3 tons of SCB waste being produced after extracting juice from 1 ton of sugarcane [13] and the SCB end use is typically to generate electricity based on incineration. Using SCB as feedstock for P(3HB) production is economically feasible, sustainable, and eco-friendly, reduces production costs, and does not compete as a food source. However, additional pretreatment steps are required to improve the release of fermentable sugars when using SCB as feedstock (for a review of pretreatment techniques, see [11]). SCB has been intensively studied as an alternative carbon source for P(3HB) production by several microorganisms, including *Brevibacterium casei*, *Burkholderia cepacian*, *Lysinibacillus* sp., and *Priestia megaterium* (formerly *Bacillus megaterium*) [2,8,10]. In addition to finding more economical substrates to reduce production costs, the co-production of other valuable products alongside P(3HB) through a single fermentation process may improve the overall economics. Several studies have reported the co-production of P(3HB) and other products such as bioethanol [14], coenzyme Q10 (CoQ10) [15], ectoine [16], inulin [17], isobutanol [18], levan polysaccharide [17,19], and violacein pigment [20]. However, very few studies have investigated co-production of P(3HB) and other products using SCB as feedstock. For example, Priya et al. [21] reported the co-production of P(3HB) (12.3 g/L, 63% of dry cell weight (DCW)) and carotenoids (4.3 g/L) by *Bacillus chungangensis* using hot water hydrolysis pretreatment of SCB as a carbon source.

*Bacillus cereus*, a Gram-positive, rod-shaped, endospore-forming bacterium, is a member of the *B. cereus* group. *B. cereus* is the most predominant species in the genus *Bacillus* and is found in diverse environments such as soil, sediments, plant roots, dust, and water [22]. The pathogenicity of *B. cereus* is strain-dependent. Some *B. cereus* strains are foodborne pathogens that cause vomiting and diarrhea, whereas others are non-pathogenic and considered safe to use as probiotics [22]. *B. cereus* is a non-fastidious bacterium that grows well in a wide variety of carbon sources and converts them to biopolymers [23]. Numerous strains of *B. cereus* have been reported to produce P(3HB) using different feedstocks, including lignocellulosic wastes (for a review, see [23]). In addition to P(3HB) production, *B. cereus* is one of the two members of the *B. cereus* group that have been identified as major producers of bacteriocins [24,25]. Several bacteriocins, such as bacicyclicin, bicereucin, cerecidin, cereucin, and thiocillin, have been reported to be produced by *B. cereus* and to contain antibacterial activity against various microorganisms, including Gram-positive and Gram-negative bacteria (for a review, see [24]).

Previously, we screened 602 local *Bacillus* isolates and obtained 79 isolates that produced P(3HB) on nutrient agar (NA) medium supplemented with 5% sucrose [26]. In the current work, these 79 P(3HB)-producing isolates were further studied for their ability to synthesize P(3HB) using sugars from the hydrolysate of SCB as feedstock. In addition, co-production of P(3HB) and bacteriocin was investigated in the current work. This is the first report describing the co-production of P(3HB) and bacteriocin by *B. cereus* using sugar from the hydrolysate of SCB as feedstock.

## 2. Materials and Methods

### 2.1. Bacteria

In total, 79 *Bacillus* isolates that had previously demonstrated production of P(3HB) in a commercial medium [26] were recovered from glycerol stocks of the culture collection of the Microbial Biotechnology Unit, Department of Science and Bioinnovation, Faculty of Liberal Arts and Science, Kasetsart University, Kamphaeng Saen Campus, Nakhon Pathom, Thailand, and streaked onto NA medium. After 24 h of incubation at 30 °C, a single colony was picked, restreaked on NA, and used in subsequent steps. *Escherichia coli* TISTR 780, *Shigella dysenteriae* DMST 1511, *Staphylococcus aureus* TISTR 746, and *Bacillus cereus* TISTR 687 were used as test microorganisms for antibacterial activity analysis.

### 2.2. Sugarcane Bagasse Hydrolysate Preparation

Sugarcane bagasse (SCB) was obtained from a local market in Nakhon Pathom province, Thailand. The collected biomass was dried in sunlight, cut into small pieces, and pulverized using a blender. Then, the biomass was sieved to a particle size of approximately 2 mm and further dried in a hot-air oven overnight at 55 °C. The chemical composition of the SCB was determined following the report by Supmeeprom et al. [27]. Alkaline pretreatment was conducted as follows: 10 g of SCB was added to 100 mL of 2% (*w*/*v*) NaOH and incubated in a water bath at 80 °C for 3 h. The pretreated SCB was separated using filtration through cheesecloth and washed with tap water until the pH decreased to 7.0; then, the product was dehydrated in the hot-air oven at 55 °C until a constant weight was recorded. To convert the LCB into fermentable sugars, 10 g of pretreated SCB was hydrolyzed through incubation with 100 mL of 50 mM citrate buffer (pH 4.8) containing 150 filter paper units of Accellerase1500 (Genencor, Rochester, NY, USA), a lignocellulolytic enzyme cocktail. After 24 h of incubation at 50 °C, the hydrolysate was passed through Whatman No. 1 filter paper, neutralized, and analyzed for its total reducing sugar (TRS) concentration using the 3,5-dinitrosalicylic acid method [28]. The SCB hydrolysate was utilized for culture medium preparation. Sugar compositions of hydrolysate were measured using a Water 600E high-performance liquid chromatography (HPLC) system (Waters Corp; Milford, MA, USA) equipped with a refractive index detector, according to another report [29].

### 2.3. Screening of P(3HB)-Producing Bacteria

Preliminary screening for P(3HB)-producing isolates was performed using nitrogen-deficient agar medium according to Thammasittirong et al. [26] with a modification of substituting the carbon source with 1.0% TRS from SCB hydrolysate. In brief, 33 mL of SCB hydrolysate (from 30.2 g/L TRS) was added to nitrogen-deficient agar medium containing 0.25% peptone, 0.25% yeast extract, 0.02% MgSO_4_, 0.01% NaCl, 0.05% KH_2_PO_4_, and 1.5% agar. The pH was then adjusted to 7.0 and distilled water was added to a final volume of 100 mL. The medium was sterilized by autoclaving at 110 °C for 20 min, then poured into Petri dishes. For Sudan black B staining, 79 P(3HB)-producing isolates were streaked on the SCB hydrolysate medium and cultivated at 30 °C for 48 h and analyzed by staining their colonies and cells with Sudan black B according to reported methods [26,30]. For Nile red fluorescent-based screening, a single colony of 79 P(3HB)-producing *Bacillus* isolates was streaked on the nitrogen-deficient agar medium containing 1.0% TRS from SCB hydrolysate as the sole carbon source and 0.5 μg/mL Nile red (dissolved in dimethyl sulfoxide). After 48 h of incubation at 30 °C, bacterial colonies were exposed to ultraviolet (UV) radiation at a wavelength of 395 nm. Positive P(3HB) accumulation colonies for Nile red screening resulted in fluorescent orange, red, or pink colonies. *Escherichia coli* was used as a negative control.

### 2.4. Quantitative Analysis of P(3HB) Production

Quantitative analysis of P(3HB) production was performed according to a published method, with some modifications [31]. Briefly, the selected P(3HB)-producing isolates were cultivated in 50 mL nitrogen-deficient medium (without agar) containing 1.0% TRS from SCB hydrolysate as the carbon source (prepared as mentioned above) for 48 h at 30 °C, with shaking at 150 rpm. The P(3HB)-accumulating bacterial cells were precipitated via centrifugation at 7500× *g* for 10 min. The cell pellet was resuspended in 10 mL of 4% sodium hypochlorite and incubated at 37 °C for 2 h. The completeness of cell hydrolysis was checked under a light microscope. Then, the lysate was centrifuged at 12,000× *g* for 20 min, after which the pellet was washed with diH_2_O and additionally washed twice with a mixture of acetone, methanol, and diethyl ether (1:1:1). The extracted P(3HB) granules were solubilized in boiling chloroform and then left to evaporate in a fume hood. P(3HB) was converted to crotonic acid by the addition of 5 mL of sulfuric acid and boiled at 100 °C for 10 min. The P(3HB) concentration was analyzed by measuring the absorbance at 235 nm using a GENESYS 10S UV-Vis (Thermo Scientific; Waltham, MA, USA) and comparing it with a standard curve of crotonic acid.

DCW and P(3HB) accumulation were analyzed according to a published method [26] in which the percentage of P(3HB) accumulation was the percentage composition of P(3HB) present in the DCW.

### 2.5. Identification of the Most Potent P(3HB)-Producing Isolate

A single colony of the most potent P(3HB)-producing isolate, using SCB hydrolysate as a carbon source, was inoculated in Luria-Bertani broth and cultivated overnight at 30 °C, with shaking at 150 rpm. Genomic DNA was extracted and purified using a Biofact Genomic DNA extraction kit (Biofact; Daejeon, Republic of Korea). The 16S rDNA gene was amplified using the 27F and 1492R universal primers, while the *gyrB* gene was amplified using the UP-1 and UP-2r primers, according to a published report [32]. The conditions for PCR amplification of the 16S rDNA and *gyrB* genes were according to Ketsakhon et al. [33]. PCR products were purified using a GF-1 PCR Clean-Up Kit (Vivantis; Shah Alam, Malaysia) and submitted for nucleotide sequencing (U2Bio; Seoul, Republic of Korea). The obtained sequences were submitted to the GenBank database and were compared with other sequences using a BLASTN search. A phylogenetic tree was generated in MEGA11 based on the *gyrB* nucleotide sequences using a neighbor-joining method with 1000 bootstrap replicates [34].

### 2.6. Optimization of Culture Medium for P(3HB) Production

The most potent P(3HB)-producing isolate was selected to study the effects of carbon and nitrogen sources on P(3HB) production. For the carbon sources, TRS from SCB hydrolysate at concentrations of 0.25%, 0.5%, 1.0%, 1.5%, or 2.0% were used to substitute the carbon source of the nitrogen-deficient medium (pH 7.0), according to Thammasittirong et al. [26]. In brief, 50 mL of SCB hydrolysate medium was prepared by adding SCB hydrolysate at final TRS concentrations of 0.25%, 0.5%, 1.0%, 1.5%, or 2.0% to nitrogen-deficient medium containing 0.25% peptone, 0.25% yeast extract, 0.02% MgSO_4_, 0.01% NaCl, and 0.05% KH_2_PO_4,_ then adjusting the pH to 7.0 and adding distilled water to a final volume of 50 mL. The medium was sterilized by autoclaving at 110 °C for 20 min. A commercial nutrient broth supplemented with 1% sucrose was used for comparison. The most potent P(3HB)-producing isolate was inoculated (2% inoculum) into each medium and cultivated for 48 h at 30 °C, with shaking at 150 rpm. After cultivation, the production of P(3HB) was analyzed using the above-mentioned method. The concentration of TRS from SCB hydrolysate in the medium that provided the highest P(3HB) production was selected to study the effect of the nitrogen source.

Five nitrogen sources (peptone, yeast extract, urea, ammonium sulfate, and ammonium nitrate) at concentrations of 0.25% and 0.5% (*w*/*v*), were used to study the effect of nitrogen on P(3HB) production. Each nitrogen source was used to substitute peptone and yeast extract in the nitrogen-deficient medium. The most potent P(3HB)-producing isolate was inoculated (2% inoculum) into the medium containing the optimum concentration of SCB hydrolysate as the carbon source and each of the nitrogen sources. After cultivation for 48 h at 30 °C, with shaking at 150 rpm, P(3HB) production was analyzed using the method mentioned above.

### 2.7. Transmission Electron Microscopy (TEM)

The most potent P(3HB)-producing isolate was cultivated in 5 mL of optimum medium containing 1.5% TRS from SCB hydrolysate as the carbon source and 0.25% yeast extract as the nitrogen source. After incubating at 30 °C for 48 h, 1 mL of the cell suspension was centrifuged, after which the cell pellet was washed with 0.1 M phosphate buffer (pH 7.0). Then, it was fixed for 2 h at 4 °C in 2.5% glutaraldehyde in 0.1 M phosphate buffer (pH 7.0). After washing three times in the same buffer, the sample was post-fixed with 1% osmium tetraoxide for 2 h at 4 °C and dehydrated in a gradient of ethanol (30%, 50%, 70%, 80%, 90%, and 100%) with each step lasting 10 min. Subsequently, the sample was settled in propylene oxide and Spurr’s resin at a ratio of 1:1 and transferred to 100% Spurr’s resin. After solidifying at 70 °C in a dry oven for 24 h, ultrathin sections were obtained using an EM UC7 Ultramicrotome (Leica; Vienna, Austria). Finally, the sections were stained with uranyl acetate and Reynold’s lead citrate and then examined using an HT-7700 TEM (Hitachi; Tokyo, Japan) at 80 kV.

### 2.8. P(3HB) Film Preparation

The P(3HB) polymer was prepared by cultivating the most potent P(3HB)-producing isolate in a 200 mL optimum medium containing 1.5% TRS from SCB hydrolysate as the carbon source and 0.25% yeast extract as the nitrogen source for 48 h at 30 °C, with shaking at 150 rpm. The P(3HB)-accumulating bacterial cells were harvested and extracted to obtain P(3HB) using the method mentioned above. Then, the extracted P(3HB) granules were solubilized in boiling chloroform and poured into a glass Petri dish, followed by evaporation in a fume hood to form a P(3HB) film. Next, the P(3HB) film was dried overnight in a hot-air oven at 50 °C and used for further characterization.

### 2.9. Characterization of the Extracted P(3HB) Polymer

#### 2.9.1. Nuclear Magnetic Resonance (NMR) Spectroscopy

The chemical structure of the extracted P(3HB) was determined using proton nuclear magnetic resonance (^1^H NMR) spectroscopy. A sample of approximately 3 mg of P(3HB) film was dissolved in 1 mL of deuterated chloroform (CDCl_3_), and the ^1^H NMR spectra were recorded at 500 MHz using an AVANCE NEO 500 MHz spectrometer (Bruker; Billerica, MA, USA) at 25 °C.

#### 2.9.2. Fourier Transform Infrared (FTIR) Spectroscopy

The functional groups in the P(3HB) film were identified using a KBr pellet method with a VERTEX 70 FTIR spectrophotometer (Bruker; Bremen, Germany). Scans were conducted under the following conditions: spectral range 4000–400 cm^−1^, 64 scans, and resolution 4 cm^−1^.

### 2.10. Antibacterial Activity Assay

The most potent P(3HB)-producing isolate was tested for antibacterial activity against *B. cereus*, *E. coli*, *S. aureus*, and *Sh. dysenteriae* using the method by Bonhi and Imran [35], with some modifications. Briefly, the most potent isolate was point-inoculated on tryptic soy agar medium and incubated for 24 h at 30 °C. Then, the plate was overlaid with 10 mL of soft NA agar (0.8%), which had been seeded with approximately 10^7^ cells of each indicator strain, before further incubation at 30 °C for 24 h. The antibacterial activity was determined by analyzing the inhibition zones surrounding colonies [35].

The antibacterial activity of the cell-free supernatant (CFS) was determined using agar well diffusion assay according to a published report [35]. The CFS was obtained from the step of P(3HB) polymer preparation from the most potent P(3HB)-producing isolate cultured in the optimum medium. After harvesting the cell via centrifugation at 7500× *g* for 10 min, the supernatant was filtered through a 0.22 µm pore size membrane. A cell suspension of test microorganisms, visually equivalent in turbidity to 0.5 McFarland standards (approximately 10^8^ CFU/mL), was prepared and swabbed on the surface of the NA agar medium. Wells were created using a sterile cork borer (diameter 6 mm) and then filled with 50 µL of CFS. After incubation at 30 °C for 24 h, inhibition zones around wells were determined.

For the antibacterial activity of the extracted P(3HB) polymer, the thin film was cut into pieces approximately 1 × 1 cm in size and sterilized using UV radiation at a wavelength of 254 nm on both sides for 30 min each. The films were placed on an NA agar medium that had been pre-swabbed with a cell suspension of the test microorganisms. After incubation for 24 h at 30 °C, antibacterial activity was determined by measuring the inhibition zones.

### 2.11. Effects of Temperature and Proteolytic Enzymes on Bacteriocin-like Substances

The bacteriocin-like substances in CFS, which had been filtered through a 0.22 µm pore-size membrane, was analyzed for stability to heat and proteolytic enzymes. To determine its thermal sensitivity, the CFS was heated to 40 °C, 60 °C, 80 °C, 100 °C, or 121 °C for 30 min. For sensitivity to proteolytic enzymes, the CFS was treated with 1 mg/mL TPCK-treated trypsin (Thermo Fisher Scientific; Waltham, MA, USA) or 1 mg/mL proteinase K (Thermo Fisher Scientific; Waltham, MA, USA) at 37 °C for 2 h, followed by enzyme inactivation through incubation at 100 °C for 10 min. Then, residual antibacterial activity was determined by comparing it with an untreated control using the agar well diffusion assay, as described above.

### 2.12. Statistical Analysis

The data were analyzed using a one-way analysis of variance followed by Tukey’s post-hoc test using the SPSS 16.0 statistical package (SPSS Inc.; Chicago, IL, USA). Values with *p* < 0.05 were considered significantly different.

## 3. Results

### 3.1. Screening of P(3HB)-Producing Bacteria

**Figure 1:**
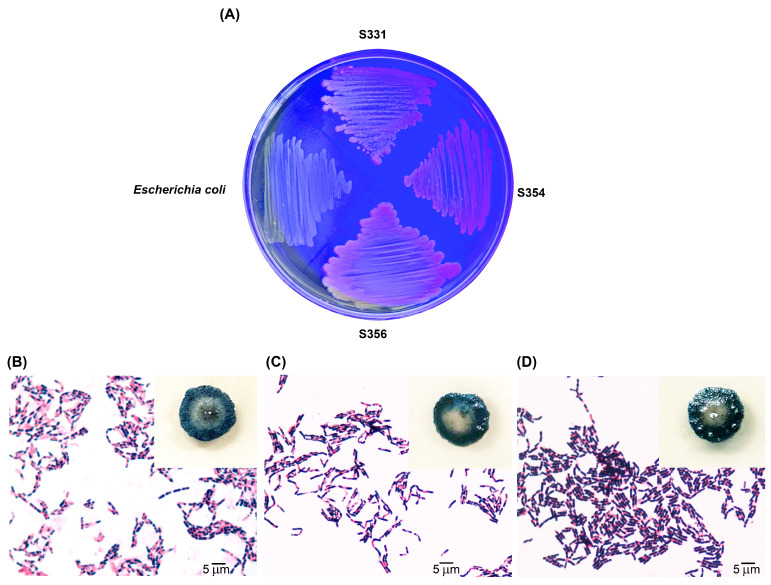
High-potential, P(3HB)-producing bacteria cultivated on nitrogen-deficient agar medium containing 1.0% total reducing sugar (TRS) from sugarcane bagasse (SCB) hydrolysate as the sole carbon source, with Nile red dye (0.5 mg/mL) showing fluorescent colonies of isolates S331, S354, and S356 under UV light (*E. coli* was used as a negative control) (**A**); and colonies and cells stained with Sudan black B after cultivation on nitrogen-deficient agar medium containing 1% TRS from SCB hydrolysate as the sole carbon source of isolates S331 (**B**); S354 (**C**); and S356 (**D**).

We previously screened 602 local *Bacillus* isolates and obtained 79 P(3HB)-producing isolates using commercial NA medium supplemented with 5% sucrose as a carbon source [26]. In the current study, these 79 *Bacillus* isolates were further tested for their abilities to synthesize P(3HB) using SCB hydrolysate as a carbon source. Analysis of the chemical composition of SCB revealed that the contents of cellulose, hemicellulose, and lignin were 41.25, 29.85, and 14.69%, respectively. The SCB hydrolysate, prepared using alkaline pretreatment and enzyme hydrolysis, contained TRS at a concentration of 30.2 g/L. HPLC analysis revealed that the hydrolysate contained glucose, xylose, and arabinose at concentrations of 15.8, 3.1, and 0.4 g/L, respectively. The SCB hydrolysate was used as the sole carbon source for the nitrogen-deficient medium preparation. After cultivation for 48 h at 30 °C in a nitrogen-deficient agar medium containing 1.0% TRS from SCB hydrolysate and 0.5 mg/mL of Nile red (lipid-specific dye), 3 of the 79 isolates (S331, S354, and S356) showed relatively high P(3HB) production, as indicated by pinkish fluorescence in their colonies under exposure to UV light (Figure 1A). Sudan black B staining showed blue-black colored colonies and large blue-black granules inside their cells (Figure 1B–D). Quantitative analysis revealed that isolate S356 produced the highest amount of P(3HB) (2.1 g/L, 58.3% of DCW), followed by isolates S354 (1.6 g/L, 45.7% of DCW) and S331 (1.1 g/L, 36.7% of DCW).

### 3.2. Identification of The Most Potent P(3HB)-Producing Isolate

**Figure 2:**
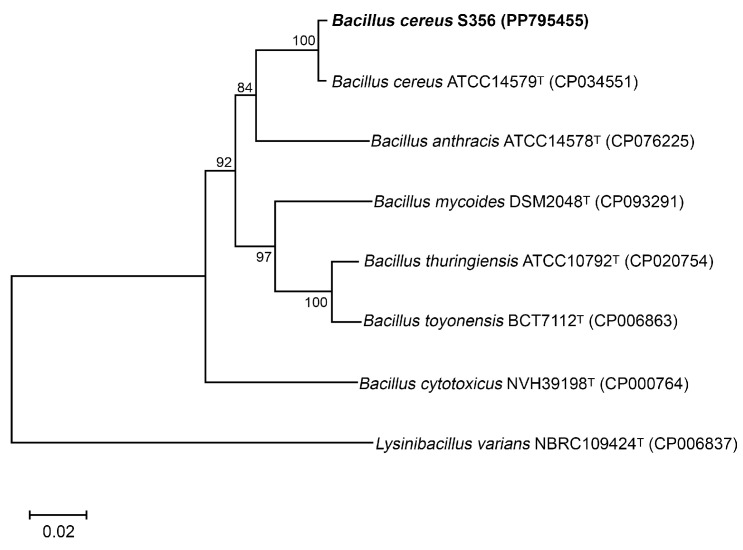
Neighbor-joining phylogenetic tree based on the *gyrB* gene of *B. cereus* S356 and closely related species, with *Lysinibacillus varians* as the outgroup. The GenBank accession numbers are indicated in parentheses. Numbers at the nodes represent percentages of bootstrap values obtained by repeating the analysis 1000 times to generate a majority consensus tree.

The isolate S356, which showed high potential for P(3HB) production using sugars from SCB hydrolysate as a carbon source, was identified based on 16S rDNA and *gyrB* gene sequences; the nucleotide sequences of the 16S rDNA and *gyrB* genes were submitted to the NCBI database (Accession numbers PP789696 and PP795455, respectively). BLASTN results using the nucleotide sequences of the 16S rDNA and *gyrB* genes revealed that isolate S356 was closely related to *Bacillus cereus* ATCC14579. A phylogenetic tree constructed based on the nucleotide sequences of the *gyrB* gene of isolate S356 and type strains of closely related species of the genus *Bacillus* is shown in Figure 2.

### 3.3. Optimization of Culture Medium for P(3HB) Production by the Most Potent B. cereus S356

**Figure 3:**
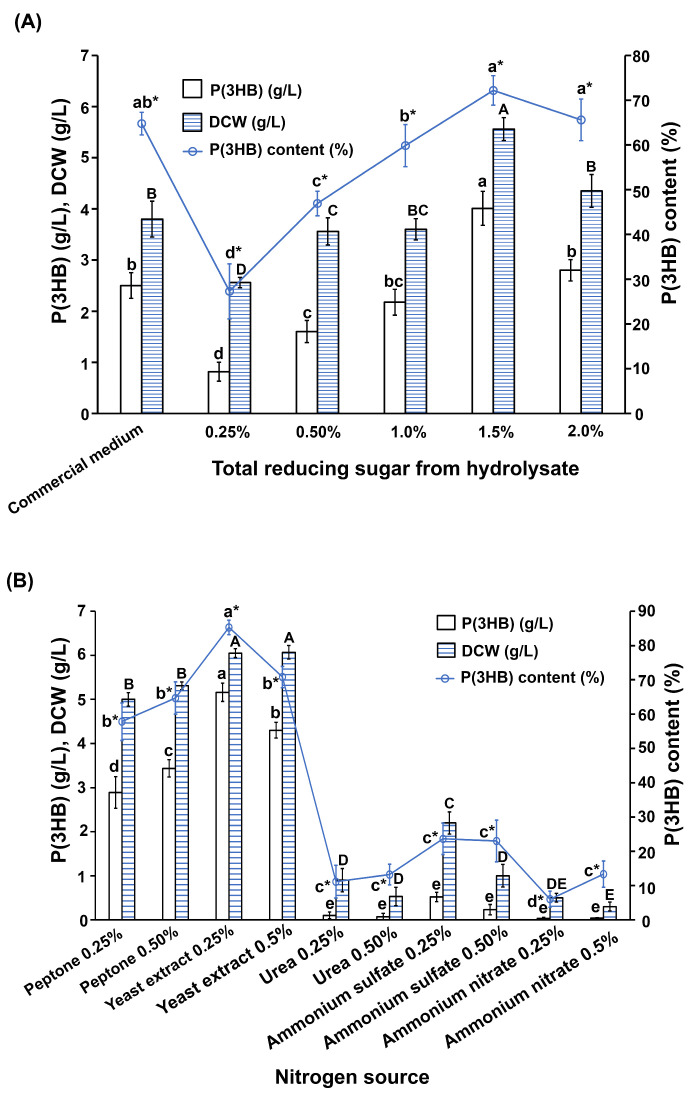
P(3HB) production by the most potent *B. cereus* S356 in (**A**) different concentrations of total reducing sugar from sugarcane bagasse as carbon sources, with commercial nutrient broth medium supplemented with 1% sucrose used for comparison; and (**B**) different nitrogen sources. Data represents mean values ± SD from three independent experiments. Different lower-case letters indicate a significant difference among different levels of P(3HB), different capital letters indicate a significant difference among different levels of DCW, and different lower-case letters with an asterisk (*) indicate a significant difference among different levels of P(3HB) content.

A study on the effects of carbon sources on P(3HB) production by the most potent *B. cereus* S356 revealed that the highest P(3HB) production (4.01 g/L, 72.1% of DCW) was achieved by cultivating *B. cereus* S356 in a medium containing 1.5% SCB hydrolysate as the carbon source (Figure 3A), which was higher than cultivation using a commercial nutrient broth medium supplemented with 1% sucrose (2.50 g/L, 65.8% of DCW)). Increasing the concentration of SCB hydrolysate from 0.25% to 1.5% resulted in increased P(3HB) production (from 0.82 g/L to 4.01 g/L), while a concentration of 2.0% led to a decrease in P(3HB) production (2.80 g/L, 64.4% of DCW), as shown in Figure 3A. Therefore, a SCB hydrolysate concentration of 1.5% was selected for further analysis of the nitrogen source.

Analysis of the effects of nitrogen sources on P(3HB) production by *B. cereus* S356 showed that only peptone and yeast extract were suitable for use as a nitrogen source, and the other three were not suitable for growth and P(3HB) production (Figure 3B). The highest P(3HB) yield (5.16 g/L, 85.3% of DCW) was obtained using 0.25% yeast extract as the nitrogen source (Figure 3B and Table 1). It was observed that increasing the concentration from 0.25% to 0.5% resulted in decreased P(3HB) production with the yeast extract, but not with the peptone, where P(3HB) production increased with increased concentration (Figure 3B).

### 3.4. Characterization of The Most Potent Isolate and Its Extracted Polymer

**Figure 4:**
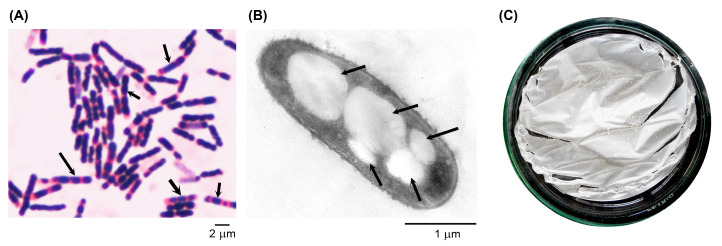
*B. cereus* S356 after 48 h of growth in optimum medium containing 1.5% of TRS from sugarcane bagasse hydrolysate and 0.25% yeast extract: (**A**) stained with Sudan black B and Safranin O and observed under a light microscope; (**B**) analyzed under transmission electron microscopy using 80 kV; and (**C**) extracted P(3HB) polymer prepared as a thin film on a glass Petri dish. Arrows indicate examples of P(3HB) granules inside the *B. cereus* cells.

After 48 h of cultivation of the highest potential P(3HB)-producing *B. cereus* S356 in the optimum broth medium containing 1.5% TRS from SCB hydrolysate and 0.25% yeast extract, the accumulation of P(3HB) in the S356 isolate was further characterized using Sudan black B staining, TEM analysis, and P(3HB) polymer extraction. The results revealed that the *B. cereus* S356 isolate accumulated a high amount of P(3HB) granules, which stained blue-black in the cells with Sudan black B (Figure 4A). Some cells accumulated higher amounts of P(3HB) granules and showed relative swelling compared to others. TEM analysis confirmed the production of P(3HB) granules in *B. cereus* S356, revealing approximately 3–5 granules (with sizes ranging from 0.19 × 0.51 to 0.71 × 1.06 µm) accumulated in each cell (Figure 4B). We observed that the granules were separated into two daughter cells during binary fission (Supplementary Figure S1). The P(3HB) film prepared from the extracted polymer is shown in Figure 4C.

#### 3.4.1. ^1^H NMR Analysis

**Figure 5:**
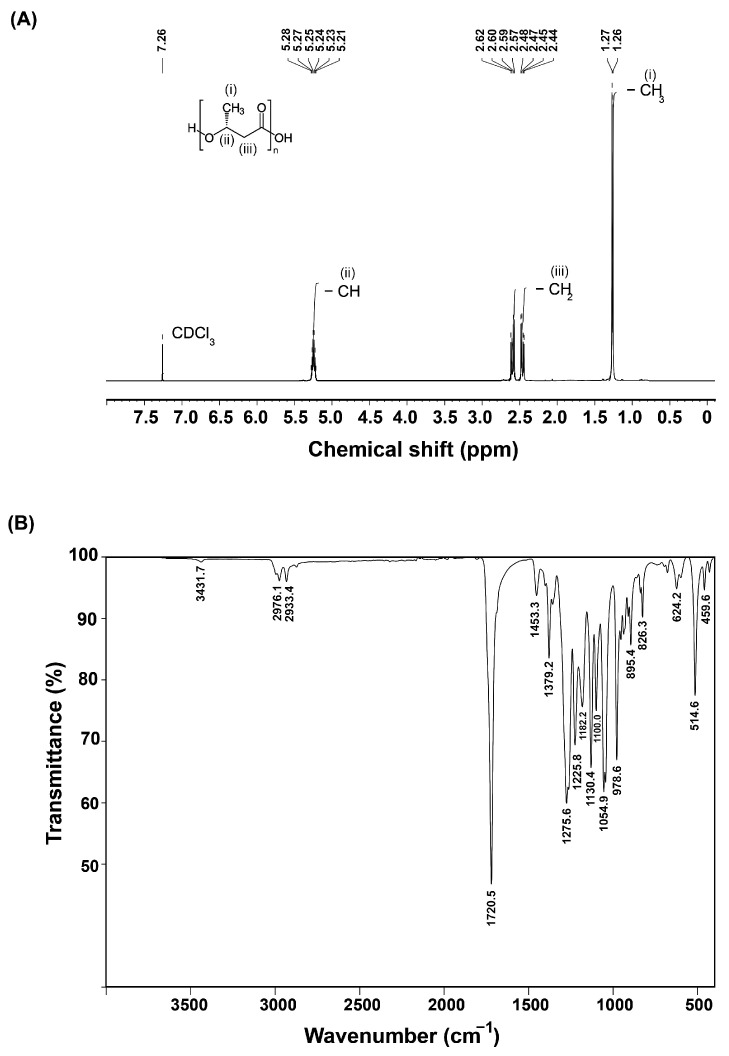
^1^H NMR spectrum (**A**) and FTIR spectrum (**B**) of the P(3HB) polymer extracted from *B. cereus* S356 cultivated in optimum medium containing 1.5% TRS from sugarcane bagasse hydrolysate and 0.25% yeast extract.

The ^1^H NMR spectrum of the P(3HB) polymer extracted from *B. cereus* S356 revealed three groups of signals at 1.26–1.27, 2.44–2.62, and 5.21–5.28 ppm, corresponding to methyl (–CH_3_), methylene (–CH_2_), and methine (–CH) groups, respectively (Figure 5A) [2,5,39]. The residual non-deuterated chloroform in the deuterated solvent CDCl_3_ at 7.26 ppm was used as an internal standard.

#### 3.4.2. FTIR Analysis

The FTIR spectrum (Figure 5B) of the P(3HB) polymer extracted from *B. cereus* S356 revealed a strong peak at 1720.5 cm^−1^ corresponding to ester group carbonyl (C=O) stretching [2,8,39]. Peaks at 2976.1 and 2933.4 corresponded to asymmetric and symmetric stretching vibrations of the methyl (–CH_3_) and methylene (–CH_2_) groups, respectively [39]. A minor peak at 3431.7 corresponded to the terminal –OH stretching vibrations [39]. These peaks, along with other peaks in the fingerprint region from 1500 cm^−1^ to 500 cm^−1^, were similar to those reported for commercial P(3HB) [8].

### 3.5. Antibacterial Activity Assays

**Figure 6:**
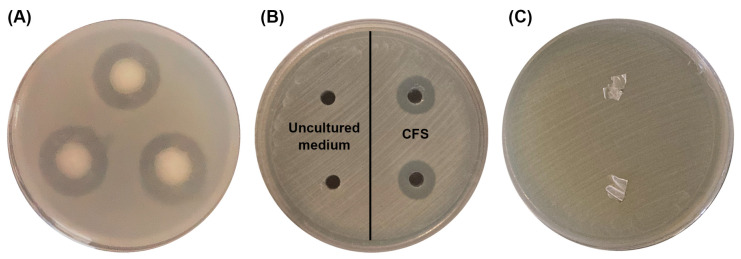
Antibacterial activity assay against *B. cereus* of (**A**) *B. cereus* S356; (**B**) cell-free supernatant (CFS) of *B. cereus* S356 after cultivation in optimum sugarcane bagasse (SCB) hydrolysate medium (**right**) and uncultured SCB hydrolysate medium as control (**left**); and (**C**) P(3HB) films prepared from polymer extracted from *B. cereus* S356.

The antibacterial activities of the most potent P(3HB)-producing bacterium, *B. cereus* S356, were assayed against four pathogenic bacteria: *B. cereus*, *E. coli*, *S. aureus*, and *Sh. dysenteriae*. The results revealed that *B. cereus* S356 only inhibited the growth of the same species, *B. cereus*, as inhibition zones were observed around colonies (Figure 6A). The CFS obtained from the cultivation of *B. cereus* S356 in the optimum medium for P(3HB) production also showed inhibition of *B. cereus* growth (Figure 6B, right-hand side), while the uncultured hydrolysate medium showed no activity (Figure 6B, left-hand side), implying that the antibacterial activity was not caused by the SCB hydrolysate composition in the medium. As expected, the extracted P(3HB) polymer prepared as thin films showed no antibacterial activity (Figure 6C).

The stability of bacteriocin-like substances in CFS after treatment with heat and proteolytic enzymes was analyzed. The results revealed that bacteriocin-like substances from *B. cereus* S356 were heat-stable and retained their activities at up to 80 °C for 30 min (Supplementary Figure S2A). Proteolytic enzyme sensitivity assays showed that bacteriocin-like substances in CFS retained their activity after treatment with trypsin for 2 h; however, there was an observed loss of antibacterial activity after treatment with proteinase K (Supplementary Figure S2B).

## 4. Discussion

The second generation of sustainable bioplastic production mainly relies on new potential sources. LCB is an attractive carbon source for producing P(3HB) bioplastics, not only because it avoids competition with the food industry for the same raw materials but also because it increases the value of agro-industrial byproducts. Previously, we screened 602 local *Bacillus* isolates and obtained 79 P(3HB)-producing isolates using a commercial medium [26]. In the current study, the 79 P(3HB)-producing bacteria were screened for their abilities to produce P(3HB) from SCB hydrolysate. Alkaline pretreatment was used for SCB pretreatment due to its efficiency in lignin removal, improved accessibility of the enzyme to cellulose, cost-effectiveness, and lower inhibitor formation [11,40]. However, using the commercial enzyme Accellerase^®^ to convert the LCB into fermentable sugars could lead to increased production costs. This limitation can be addressed by developing in-house enzymes. Isolate S356 had the highest P(3HB) production (2.1 g/L) in the medium containing 1.0% TRS of SCB hydrolysate as a carbon source, implying that this strain could utilize sugars from the hydrolysate for P(3HB) production. Increasing the TRS concentration from 0.25% to 1.5% increased P(3HB) production, but 2.0% TRS showed decreasing P(3HB) production. The decrease in P(3HB) production following cultivation using 2.0% TRS from hydrolysate may have been due to the osmotic stress affecting growth and P(3HB) production. This was consistent with Attapong et al. [2], who reported that increasing the concentration of the carbon sources could enhance P(3HB) production; however, increasing by an amount higher than the optimum concentration produced osmotic pressure, which could have negative effects on growth and P(3HB) production. In addition, our previous work reported that an increase in the concentration of the sugarcane juice carbon source to more than the optimum concentration of 2% total soluble solids resulted in the inhibition of bacterial growth and P(3HB) production, possibly due to a hypertonic effect [26]. Different sugar concentrations in the range of 1–2.5% (TRS) or 4–10% (*v*/*v*) of SCB hydrolysate have been reportedly used for P(3HB) production by different bacteria (Table 1). Studying the effects of nitrogen sources revealed similar findings to our previous report, i.e., that organic nitrogen sources, including peptone and yeast extract, were suitable; however, urea and inorganic nitrogen sources, including ammonium nitrate and ammonium sulfate, were not suitable for P(3HB) production using *B. thuringiensis* [26]. Yeast extract, which provided the highest P(3HB) production (5.16 g/L, 85.3% of DCW) in the current study, was also reported as the optimum nitrogen source for P(3HB) production with several bacteria, including *Bacillus safensis* EBT1 [36] and *Bacillus thuringiensis* [26]. Ammonium nitrate was unsuitable as a nitrogen source for P(3HB) production in the current work but was suitable for a *Bacillus* sp. in another report [37].

Isolate S356 was identified as *Bacillus cereus* based on 16S rDNA and *gyrB* gene sequences. Although the pathogenicity of *B. cereus* is strain-dependent and usually non-pathogenic [41], further characterization of *B. cereus* S356 is required. However, using purified products without cells makes it safer. Martínez-Herrera et al. [23] reported that *B. cereus* is an interesting model for producing PHAs due to its adaptive and productive properties, its ability to use residual substrates as carbon sources, and the bioplastics produced from *B. cereus* are biocompatible, nontoxic, and have low brittleness. *B. cereus* has been reported to produce P(3HB) using various lignocellulosic materials such as *Agave durangensis* leaves [42], palm empty fruit bunch [43], and grape residues [44]. SCB has been reported to be used for P(3HB) production by some strains of bacteria (Table 1). However, to our knowledge, there has been no published report on P(3HB) production by *B. cereus* using lignocellulosic SCB as a carbon source. Our results showed that *B. cereus* S356 accumulated a high amount of P(3HB) (approximately 85% of DCW), comparable to that produced using *Bacillus safensis* EBT1 and higher than for *Bacillus* sp., *B. thuringiensis* IAM 12077, *Lysinibacillus* sp., and *Priestia megaterium* KKR5 (Table 1). This may have been due to the P(3HB) production yields when using SCB hydrolysate as a carbon source varying depending on several factors such as the bacterial strain, method of SCB pretreatment, amount of hydrolysate sugar in the medium, and cultivation conditions.

^1^H NMR analysis revealed three peak signal groups corresponding to methyl (–CH_3_), methylene (–CH_2_), and methine (–CH) of the P(3HB) polymer extracted from *B. cereus* S356, which are typically reported for commercialized P(3HB) and from extraction from other microorganisms [2,5,26,45,46], indicating that the polymer extracted from *B. cereus* S356 was uncontaminated P(3HB). In addition, the FTIR spectrum exhibited functional groups and a fingerprint region similar to those of other microorganisms and commercial P(3HB) [8,39], confirming that the extracted polymer from *B. cereus* S356 was P(3HB).

Co-production is one of the attractive strategies for producing more than one product at a lower cost. Several reports have studied the co-production of P(3HB) with other products such as bioethanol [14], ectoin [16], inulin [17], isobutanol [18], levan polysaccharide [17,19], and violacein pigment [20]. However, to the best of our knowledge, there has been no published study to date reporting on the co-production of P(3HB) and bacteriocin. *B. cereus* has been reported to produce various bacteriocins, such as bacicyclicin, bicereucin, cerecidin, cereucin x, and thiocillin, which exhibit antibacterial activity against several bacteria, including the closely related species *B. anthracis*, *B. badius*, *B. cereus*, *B. circulans*, *B. coagulans*, *B. firmus*, *B. laterosporus*, *B. licheniformis*, *B. maroccanus*, *B. megaterium*, *B. mycoides*, *B. pumilus*, *B. subtilis*, *B. thuringiensis*, and *B. weihenstephanensis* [24]. In the current work, *B. cereus* S356, which produced a high P(3HB) content, showed antibacterial activity against the same species, *B. cereus*. Antibacterial activity was also found in CFS of *B. cereus* S356 cultivated in the medium containing SCB hydrolysate as a carbon source, implying that *B. cereus* S356 could produce antibacterial substances and secrete them into the supernatant even when grown in a medium containing hydrolysate. The antibacterial substances produced by *B. cereus* S356 exhibited bacteriocin properties, as they inhibited a narrow spectrum against closely related species and showed tolerance to high temperature and proteolytic enzymes [47]. Similar results were reported by Chaabouni et al. [48], who identified three cerein bacteriocins produced by *B. cereus*, which tolerated trypsin but degraded under proteinase K treatment and retained antibacterial activity against *B. cereus* ATCC11778 after exposure to high-temperature treatments of up to 80 °C. Co-production of P(3HB) and bacteriocin simultaneously by *B. cereus* S356 using the SCB hydrolysate-based medium could reduce costs by using lignocellulosic waste and reduce the total production cost by obtaining two valuable products in one fermentation. However, larger-scale fermentation should be undertaken in future studies.

## 5. Conclusions

This study investigated P(3HB) production using SCB hydrolysate as a carbon source in 79 *Bacillus* isolates previously known to produce P(3HB) in a commercial NA medium. Isolate S356, identified as *Bacillus cereus* using 16S rDNA and *gyrB* gene sequences, exhibited the highest P(3HB) production (5.16 g/L, 85.3% of DCW) in the optimal medium containing 1.5% TRS from SCB hydrolysate as a carbon source and 0.25% yeast extract as a nitrogen source. Analysis based on ^1^H NMR and FTIR confirmed that the extracted polymer from *B. cereus* S356 was P(3HB). During P(3HB) production, *B. cereus* S356 secreted bacteriocin into the culture medium. The CFS showed antibacterial activity against the same species, *B. cereus*. The antibacterial substances from *B. cereus* S356 exhibited bacteriocin properties. Overall, this study demonstrated, for the first time, the co-production of P(3HB) and bacteriocin by *B. cereus* using SCB hydrolysate as a carbon source, highlighting the possibility of co-producing two valuable products using low-cost lignocellulosic agricultural waste, which may reduce the overall production cost.

## Figures and Tables

**Table 1:** P(3HB) production by *B. cereus* S356 and other bacteria using sugar from sugarcane bagasse as the carbon source.

Bacterial Strain	Pretreatment/Hydrolysis of SCB	Cultivation Conditions	SCB Hydrolysate in Medium	P(3HB) (g/L)	Dry Cell Weight (g/L)	P(3HB) Accumulation (% of DCW)	Reference
*Bacillus cereus*	2% NaOH, 80 °C for 3 h/enzymatic hydrolysis	37 °C, 48 h	1.5% (TRS)	5.16	6.05	85.3	This study
*Bacillus safensis* EBT1	NR	45 °C, 48 h	1% (*w*/*v*)	5.92	6.56	90.2	[36]
*Bacillus* sp.	Zinc chloride/ acid hydrolysis, 70–180 °C	37 °C, 48 h	4% (*v*/*v*)	5.00	9.00	55.5	[37]
*Bacillus thuringiensis* IAM 12077	0.5–5.0% H_2_SO_4_, autoclave 121 °C, 30 min	room temperature for 48 h	10% (*v*/*v*)	4.20	10.60	39.6	[38]
*Burkholderia cepacia* ASL22	1% H_2_SO_4_, autoclave at 121 °C, 15 min	30 °C, 72 h	2.5% (TRS)	1.03	10	10.3	[2]
*Lysinibacillus* sp.	2% peracetic acid solution, autoclave 121 °C, 15 min/enzymatic hydrolysis	37 °C 48 h	2% (TRS)	3.05	6.05	50.5	[10]
*Lysinibacillus* sp.	Ultrasound, 2% NaOH, autoclave 121 °C, 15 min/enzymatic hydrolysis	37 °C 48 h	2% (TRS)	4.32	7.81	55.4	[10]
*Lysinibacillus* sp.	2%NaOH, autoclave 121 °C, 15 min/enzymatic hydrolysis	37 °C 48 h	2% (TRS)	3.54	6.88	51.5	[10]
*Priestia megaterium* KKR5	1% H_2_SO_4_, autoclave at 121 °C, 15 min	30 °C, 72 h	2.5% (TRS)	1.36	11.62	11.7	[2]

NR = not reported.

## Data Availability

The data presented in this study are available on request from the corresponding author.

## Supplementary Materials

The following supporting information can be downloaded at: https://www.mdpi.com/article/10.3390/polym16142015/s1, Figure S1: TEM images show P(3HB) granules separated into two daughter cells during binary fission, Figure S2: Antibacterial activity against *B. cereus* of bacteriocin-like substances in CFS from the *B. cereus* S356 after heating at 60 °C, 80 °C, 100 °C, and 121 °C for 30 min (A) and proteolytic cleavage by trypsin and proteinase K at 37 °C for 2 h (B).
