# Supplementary material for: Valorization of Sugarcane Bagasse for Co-Production of Poly(3-hydroxybutyrate) and Bacteriocin Using Bacillus cereus Strain S356
Source: Polymers (Basel). 2024 Jul 15;16(14):2015. doi: 10.3390/polym16142015 (PMC11281070; doi:10.3390/polym16142015)
Supplement: Supplementary file 1 [file polymers-16-02015-s001.zip › polymers-3038137-SI.pdf]

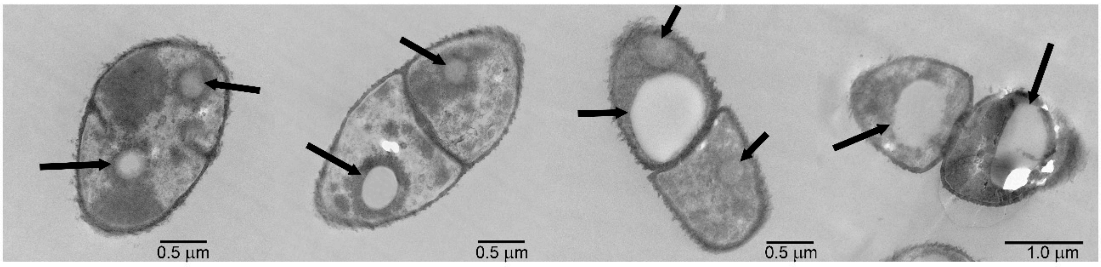

Figure S1: TEM images show PHB granules separated into two daughter cells during binary fission. Arrows indicate examples of P(3HB) granules inside the *B. cereus* cells.

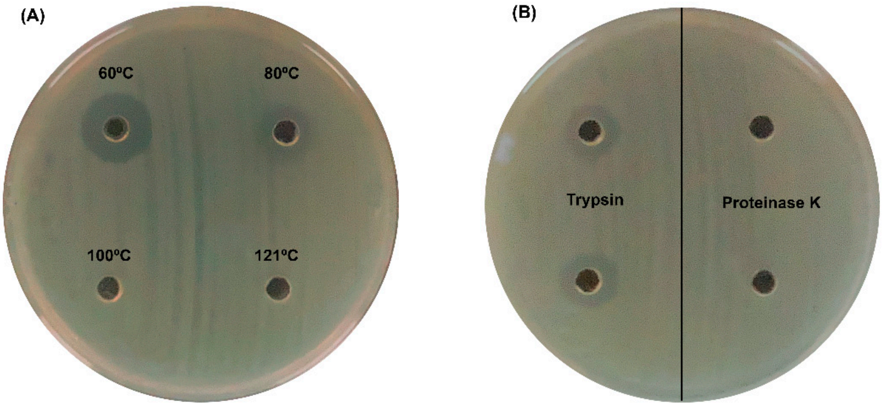

Figure S2: Antibacterial activity against *B. cereus* of bacteriocin-like substances in CFS from the *B. cereus* S356 after heated at 60°C, 80°C, 100°C, and 121°C for 30 min **(A)** and proteolytic cleavage by trypsin and proteinase K at 37°C for 2 h **(B).**
